# Supplementary material for: Genome-Wide Analysis of Sweet Potato Ammonium Transporter (AMT): Influence on Nitrogen Utilization, Storage Root Development and Yield
Source: Int J Mol Sci. 2023 Dec 13;24(24):17424. doi: 10.3390/ijms242417424 (PMC10744204; doi:10.3390/ijms242417424)
Supplement: Supplementary file 1 [file ijms-24-17424-s001.zip › ijms-2751207-supplementary.pdf]

Genome-Wide Analysis of Sweet Potato Ammonium Transporter (AMT): Influence on Nitrogen Utilization, Storage Root Development and Yield

Ya-Yi Meng [1,2,3], Ning Wang [2], Hai-Yan Zhang [4], Ran Xu [1,2,3] and Cheng-Cheng Si [1,2,3]*

[1] Collaborative Innovation Center of Nanfan and High-Efficiency Tropical Agriculture, Hainan University, Sanya 572025, China; 21220951310162@hainanu.edu.cn (Y.-Y.M.); xuran@hainanu.edu.cn (R.X.)
[2] Key Laboratory of Quality Regulation of Tropical Horticultural Crop in Hainan Province, School of Tropical Agriculture and Forestry (School of Agricultural and Rural, School of Rural Revitalization), Hainan University, Danzhou 571700, China; nwanghnu@163.com
[3] School of Breeding and Multiplication (Sanya Institute of Breeding and Multiplication), Hainan University, Sanya 572025, China
[4] Scientific Observation and Experimental Station of Tuber and Root Crops in Huang-Huai-Hai Region of Agriculture Ministry, Crop Research Institute, Shandong Academy of Agricultural Sciences, Jinan 250100, China; zhanghaiyan@saas.ac.cn
* Correspondence: ccsi@hainanu.edu.cn

Figure S1 Locations of *IbAMT* gene family members on chromosomes

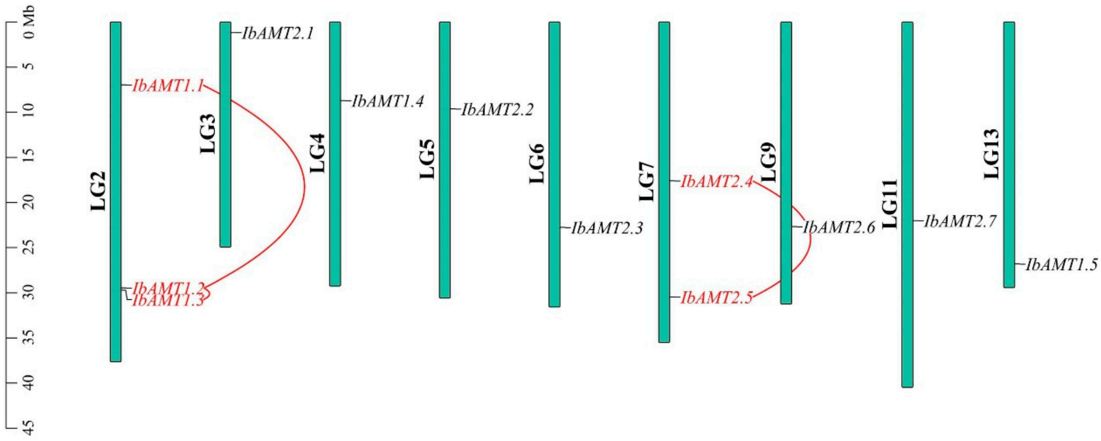

The chromosome number is listed at the left side of each chromosome, while the number to the right of each chromosome represents the location of the *IbAMT* gene on the right. The segmental duplicated genes are linked by red line.

Figure S2 It is the sequence information of motif in Fig 5

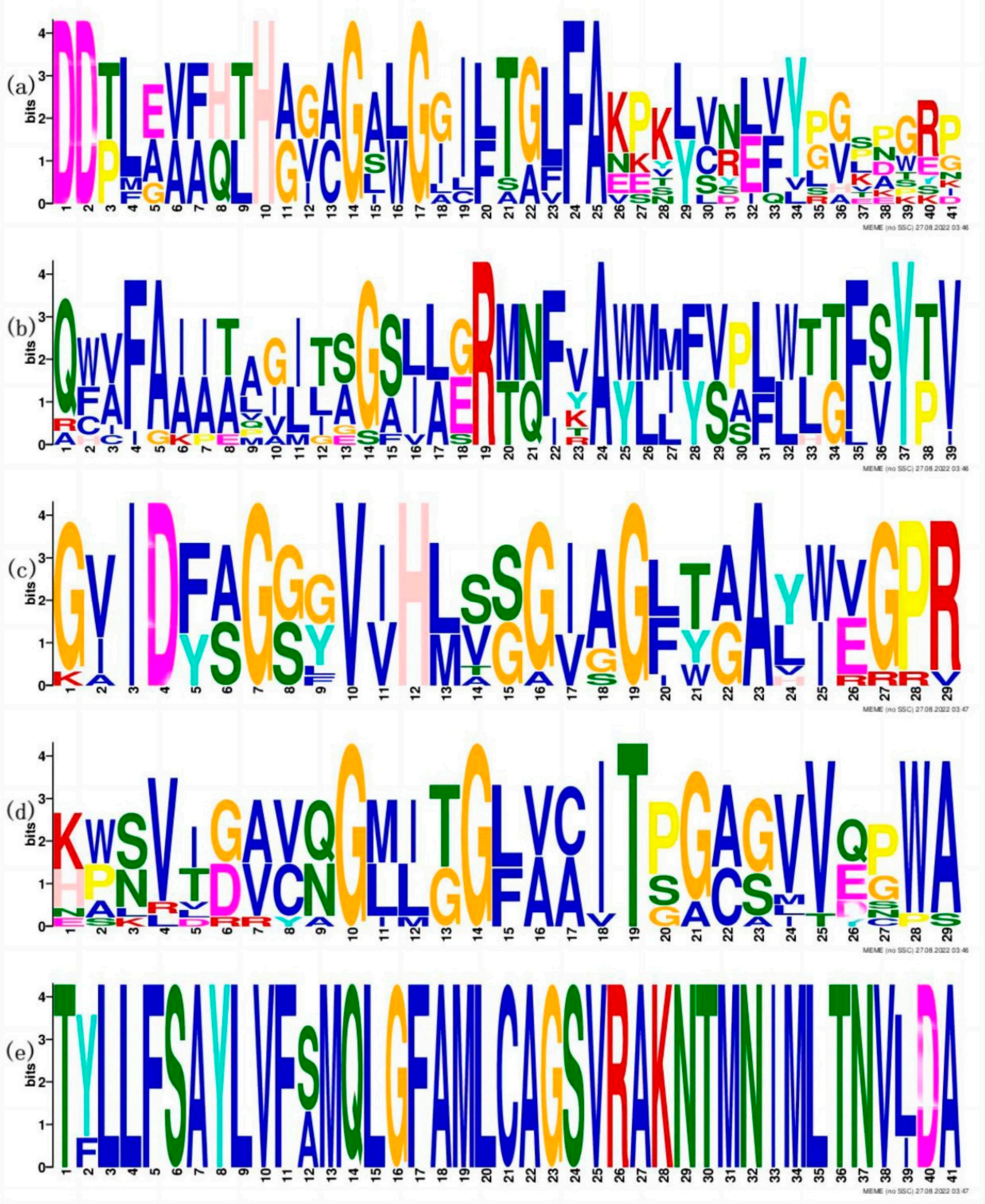

(a) motif1, (b) motif2, (c) motif3, (d) motif4, (e) motif5.

Figure S3 Cis-elements analysis of the *IbAMT* genes promoter regions

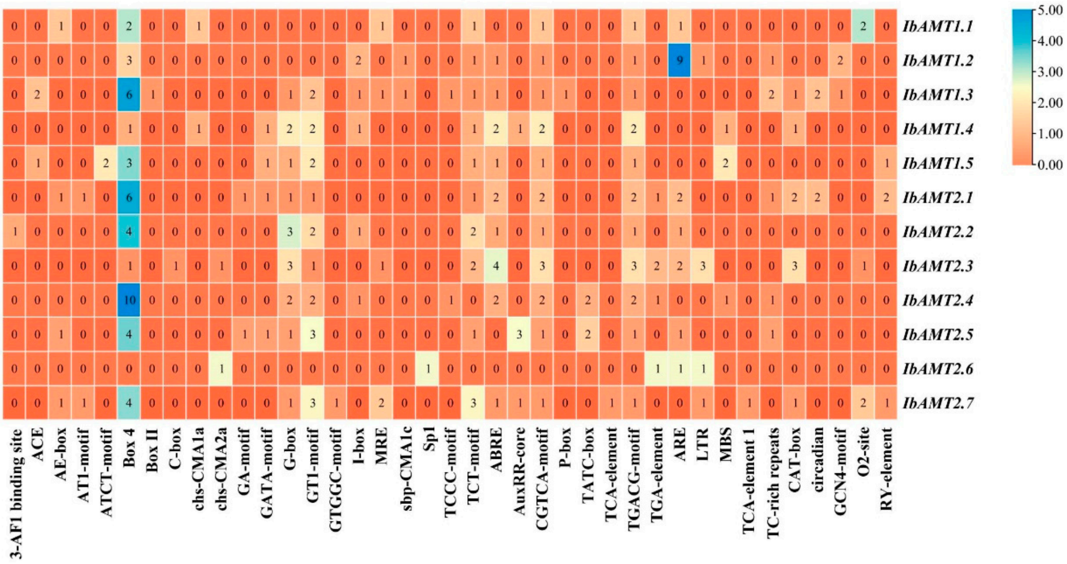

The types and numbers of potential elements in the promoter regions 2-kb upstream of the *IbAMT* genes were analyzed by PlantCARE.

Table S1 Dry matter accumulation at canopy closure and harvest

Period	Year	Treatment	Leaf dry weight per plant (g)	Petiole dry weight per plant (g)	Stem dry weight per plant (g)	Storage root dry weight per plant (g)
Canopy Closure	2021	NN	-	-	-	-
		LN	3.05±0.13 c	1.04±0.13 c	3.14±0.20 c	7.06±0.86 b
		MN	5.05±0.39 a	1.83±0.13 a	5.45±0.21 a	10.66±0.89 a
		HN	4.31±0.51 b	1.69±0.17 b	4.98±0.51 b	5.95±0.56 c
	2022	NN	1.76±0.20 d	0.61±0.04 d	1.49±0.20 d	4.43±1.23 c
		LN	3.78±0.20 c	1.66±0.12 c	4.66±0.27 c	7.31±1.28 b
		MN	6.16±0.25 a	2.61±0.16 a	7.26±0.42 a	11.11±2.38 a
		HN	4.46±0.41 b	2.00±0.10 b	5.34±0.68 b	6.59±0.60 b
Harvest	2021	NN	7.54±0.46 c	4.51±0.26 c	9.68±0.69 c	44.16±7.56 c
		LN	13.37±0.60 b	7.50±0.36 b	16.07±0.73 b	70.54±10.26 ab
		MN	15.46±0.92 a	7.51±0.41 b	16.58±0.45 b	79.84±11.51 a
		HN	15.60±0.33 a	8.88±0.29 a	18.47±0.45 a	61.49±8.37 b
	2022	NN	7.98±0.29 d	6.05±0.27 d	15.81±0.85 d	44.48±8.22 c
		LN	12.04±0.96 c	9.69±0.22 c	24.81±0.76 c	62.81±11.97 ab
		MN	15.10±0.75 b	11.49±0.30 b	29.76±0.56 b	69.82±14.37 a
		HN	16.34±1.26 a	13.55±0.57 a	32.77±1.29 a	52.16±13.36 bc

Note: NN, no N; LN, 60 kg ha^{-1} N; MN, 120 kg ha^{-1} N; HN, 180 kg ha^{-1} N, Two-way ANOVA, LSD. The values after the ± sign are the standard deviation. Values followed by lowercase letters within a column are significantly different among N treatments ($p<0.05$). *—$p<0.05$; **—$p<0.01$.

Table S2 N accumulation at canopy closure and harvest

Period	Year	Treatment	Leaf (g)	Petiole (g)	Stem (g)	Storage root (g)
Canopy closure	2021	NN	-	-	-	-
		LN	9.16 c	1.28 c	4.19 c	8.76 b
		MN	24.74 a	3.43 a	12.67 a	20.17 a
		HN	16.39 b	2.35 b	8.01 b	7.58 c
	2022	NN	3.43 d	0.66 d	1.44 d	2.15 c
		LN	12.64 c	2.63 c	5.79 c	6.20 b
		MN	31.67 a	7.46 a	14.75 a	12.50 a
		HN	21.62 b	4.92 b	9.76 b	6.41 b
Harvest	2021	NN	17.03 d	4.23 c	6.28 c	29.58 c
		LN	40.64 c	9.61 b	15.42 b	62.78 b
		MN	60.78 b	13.31 a	21.97 a	81.43 a
		HN	65.82 a	13.72 a	23.54 a	55.33 b
	2022	NN	14.57 d	3.65 d	7.83 d	12.80 c
		LN	31.65 c	8.69 c	17.36 c	24.49 b
		MN	44.08 b	12.51 b	24.10 b	32.81 a
		HN	48.85 a	15.98 a	29.49 a	23.46 b

Note: NN, no N; LN, 60 kg ha^{-1} N; MN, 120 kg ha^{-1} N; HN, 180 kg ha^{-1} N, Two-way ANOVA, LSD. Values followed by lowercase letters within a column are significantly different among N treatments ($p<0.05$). *—$p<0.05$; **—$p<0.01$.

Table S3 Similarity alignment of AMT proteins in sweet potato

Gene name	*IbAMT 1.1*	*IbAMT 1.2*	*IbAMT 1.3*	*IbAMT 1.4*	*IbAMT 1.5*	*IbAMT 2.1*	*IbAMT 2.2*	*IbAMT 2.3*	*IbAMT 2.4*	*IbAMT 2.5*	*IbAMT 2.6*	*IbAMT 2.7*
IbAMT1.1	100.00											
IbAMT1.2	68.14	100.00										
IbAMT1.3	68.35	99.60	100.00									
IbAMT1.4	69.83	82.22	82.42	100.00								
IbAMT1.5	69.83	77.80	76.67	78.59	100.00							
IbAMT2.1	14.32	13.67	13.02	14.97	15.62	100.00						
IbAMT2.2	17.11	18.22	18.22	19.11	17.78	42.00	100.00					
IbAMT2.3	17.08	18.56	18.07	15.35	18.07	50.00	42.33	100.00				
IbAMT2.4	15.19	15.63	16.67	16.88	16.67	40.78	54.00	35.64	100.00			
IbAMT2.5	15.85	17.07	15.37	16.59	17.07	40.49	50.73	47.77	42.68	100.00		
IbAMT2.6	16.21	17.13	16.82	17.43	13.15	39.76	62.69	32.11	60.24	40.37	100.00	
IbAMT2.7	18.71	16.70	16.48	20.04	19.60	55.23	52.12	55.69	50.56	53.66	53.82	100.00

Table S4 Ka/Ks ratios and estimated divergence time for paralogous *AMT* genes in sweet potato

Gene name	Gene name	Duplicated type	Ka	Ks	Ka/Ks	Types of selection	Divergence time (Mya)
IbAMT1.1	*IbAMT1.2*	tandem duplication	0.19	1.13	0.17	Purify selection	92.89
IbAMT1.2	*IbAMT1.3*	tandem duplication	0.00	0.01	0.23	Purify selection	0.65
IbAMT2.4	*IbAMT2.5*	tandem duplication	0.34	1.68	0.2	Purify selection	138.09
